# Research advances in natural sesquiterpene lactones: overcoming cancer drug resistance through modulation of key signaling pathways

**DOI:** 10.20517/cdr.2024.178

**Published:** 2025-03-24

**Authors:** Chi Teng, Jia-Wen Chen, Li-Sha Shen, Sibao Chen, Guo-Qing Chen

**Affiliations:** ^1^College of Pharmacy, Shenzhen Technology University, Shenzhen 518118, Guangdong, China.; ^2^Institute of Medicinal Plant Development, Chinese Academy of Medical Sciences and Peking Union Medical College, Beijing 100193, China.; ^3^State Key Laboratory of Chinese Medicine and Molecular Pharmacology (Incubation), The Hong Kong Polytechnic University Shenzhen Research Institute, Shenzhen 518057, Guangdong, China.; ^4^Chongqing Academy of Chinese Materia Medica, Chongqing 400065, China.; ^5^Department of Food Science and Nutrition, The Hong Kong Polytechnic University, Hong Kong 999077, China.; ^6^Research Centre for Chinese Medicine Innovation, The Hong Kong Polytechnic University, Hong Kong 999077, China.; ^#^Authors contributed equally.

**Keywords:** Sesquiterpene lactone, natural product, anticancer, drug resistance

## Abstract

Cancer remains a significant global health challenge, with current chemotherapeutic strategies frequently limited by the emergence of resistance. In this context, natural compounds with the potential to overcome resistance have garnered considerable attention. Among these, sesquiterpene lactones, primarily derived from plants in the Asteraceae family, stand out for their potential anticancer properties. This review specifically focuses on five key signaling pathways: PI3K/Akt/mTOR, NF-κB, Wnt/β-catenin, MAPK/ERK, and STAT3, which play central roles in the mechanisms of cancer resistance. For each of these pathways, we detail their involvement in both cancer development and the emergence of drug resistance. Additionally, we investigate how sesquiterpene lactones modulate these pathways to overcome resistance across diverse cancer types. These insights highlight the potential of sesquiterpene lactones to drive the advancement of novel therapies that can effectively combat both cancer progression and drug resistance.

## INTRODUCTION

Cancer remains a major global public health threat, with significant impacts on human well-being. In 2018, the World Health Organization reported that cancer was the second leading cause of death worldwide, accounting for approximately 9.6 million deaths annually^[1]^. Although significant progress has been made in treatment options, including chemotherapy, radiotherapy, surgery, immunotherapy, and targeted therapy, the effectiveness of these approaches is often compromised by the emergence of chemotherapy resistance. This resistance reduces therapeutic efficacy and frequently results in treatment failure^[2]^. Consequently, understanding and addressing the underlying mechanisms of drug resistance is crucial for advancing both experimental and clinical cancer research, highlighting the urgent need for novel anticancer agents.

Natural products derived from microorganisms, fungi, and plants have attracted significant attention as promising sources of innovative anticancer agents. These compounds are valued for their unique biological activities and chemical diversity. Notable examples such as vincristine, etoposide, topotecan, and paclitaxel have been successfully developed into clinically approved therapies^[3]^. Their appeal lies not only in their potent anticancer effects but also in their relatively lower toxicity profiles and reduced likelihood of inducing drug resistance. Moreover, many natural products are capable of overcoming multidrug resistance (MDR), further highlighting their potential in modern cancer treatment^[4]^.

Among these natural products, sesquiterpene lactones, a prominent class of secondary metabolites derived primarily from plants of the Asteraceae family, stand out for their diverse biological activities^[5]^. Characterized by a 15-carbon skeleton composed of three isoprene units and a γ-lactone group (cyclic ester), these compounds display remarkable structural diversity. This diversity underpins their antibacterial, anti-inflammatory, and anticancer properties, making them promising candidates for drug discovery^[6]^.

Emerging evidence underscores the significant anticancer potential of sesquiterpene lactones, particularly their effects on cancer cell development, proliferation, and metastasis^[7]^. These compounds modulate key signaling pathways involved in cancer progression, including apoptosis induction, cell cycle arrest, and immune response regulation within the tumor microenvironment. Notably, compounds such as artemisinin, artesunate, and parthenolide have advanced to clinical trials, further demonstrating their therapeutic potential^[8-10]^. In addition, sesquiterpene lactones have shown promise in overcoming drug resistance. For instance, xanthatin, costunolide, and micheliolide sensitize cancer cells to chemotherapeutic agents, providing innovative strategies to address this challenge^[11-13]^. These findings highlight the dual role of sesquiterpene lactones as both potent anticancer agents and modulators of drug resistance, reinforcing the need for a comprehensive review of recent research progress.

This review systematically analyzes research articles published between 2014 and 2024, from Google Scholar and PubMed, focusing on the anticancer and resistance-modulating properties of sesquiterpene lactones. After rigorous screening, 30 compounds with significant anticancer activity and potential to overcome drug resistance were identified. We first classify these compounds and present their chemical structures, natural sources, and anticancer activities. We then explore their mechanisms, focusing on five critical signaling pathways: phosphoinositide 3-kinase (PI3K)/Akt/mTOR, nuclear factor kappa-light-chain-enhancer of activated B cells (NF-κB), Wnt/β-catenin, mitogen-activated protein kinase (MAPK)/extracellular signal-regulated kinase (ERK), and signal transducer and activator of the transcription 3 (STAT3), which play pivotal roles in cancer progression and drug resistance. By elucidating these mechanisms, this review aims to offer valuable insights into the development of sesquiterpene lactones as therapeutic agents, advancing both basic and clinical research.

## CLASSIFICATION OF SESQUITERPENE LACTONES WITH ANTICANCER ACTIVITY

Recent studies have identified several classes of sesquiterpene lactones with significant anticancer properties, derived from plants such as *Artemisia annua* L*.*, *Inula helenium* L*.*, *Elephantopus scaber* L*.*, *Centipeda minima* L*.*, and *Tanacetum parthenium* L*.* These compounds exhibit remarkable structural diversity, which correlates with their distinct biological activities, making them promising candidates for anticancer drug development^[10,14-17]^. The following sections will examine the structural features and anticancer activities of key sesquiterpene lactone groups.

### Artemisinin compounds

**Figure 1:**
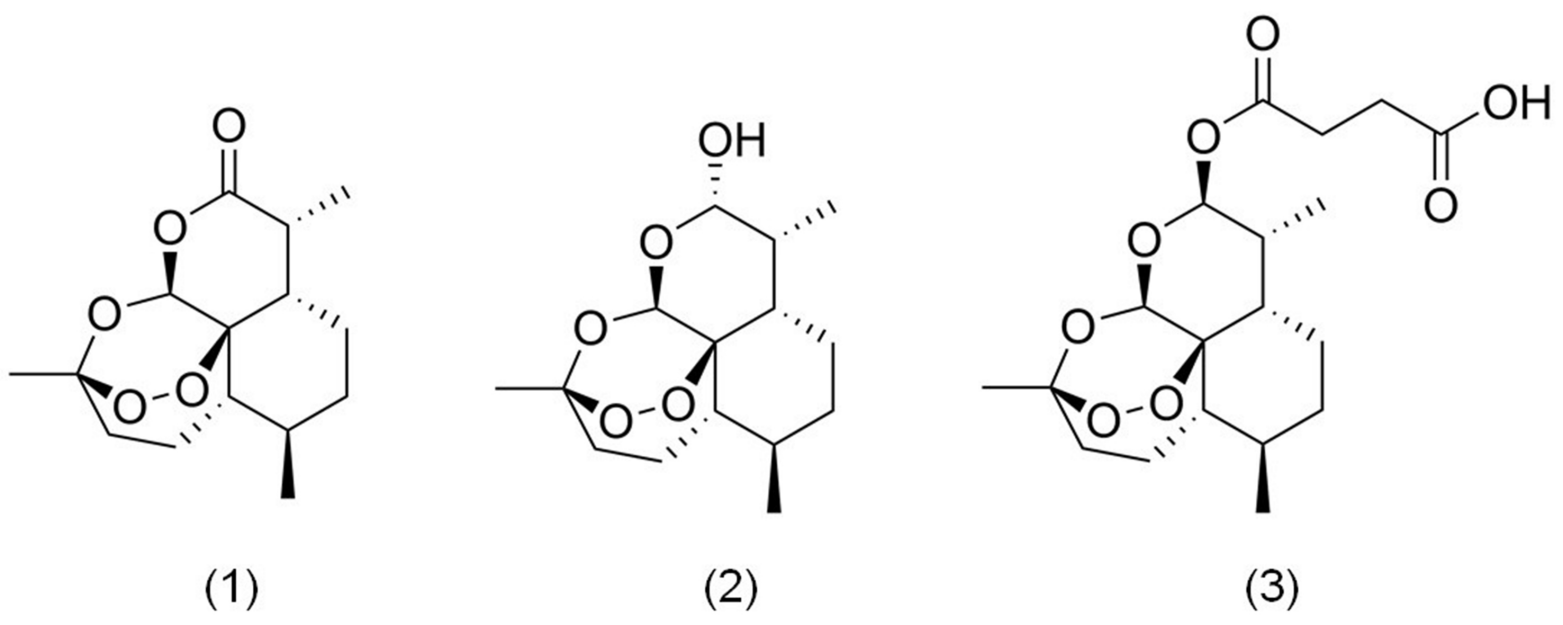
The chemical structures of artemisinin compounds. (1) Artemisinin; (2) Dihydroartemisinin; (3) Artesunate.

Sesquiterpene lactones derived from *Artemisia annua* L., collectively known as artemisinin and its derivatives, are shown in Figure 1. Artemisinin (1), a sesquiterpene lactone featuring an endoperoxide moiety, gained global recognition for its potent antimalarial activity following its isolation in the 1970s by Chinese scientist Tu Youyou^[18]^. Structurally, artemisinin is characterized by its unique peroxide bridge, lactone ring, and a rare 1,2,4-trioxane unit^[19]^, with the peroxide bond playing a crucial role in its pharmacological effects^[20]^. The discovery of artemisinin revolutionized malaria treatment, establishing it as the first-line therapy. However, its clinical application is limited by challenges such as poor bioavailability, water solubility, and a short half-life^[21]^. To overcome these limitations, derivatives with improved pharmacokinetic profiles have been developed. For example, artemisinin can be reduced to dihydroartemisinin (2) using sodium borohydride, which is then esterified with succinic anhydride to produce artesunate (3)^[22]^. These derivatives exhibit improved chemical stability and water solubility, enhancing their clinical efficacy.

Beyond its well-established antimalarial effects, artemisinin compounds have gained attention for their significant anticancer potential^[23]^. For example, Jia *et al.* demonstrated that artemisinin inhibits gallbladder cancer (GBC) cell proliferation by inducing apoptosis through increased intracellular reactive oxygen species (ROS)^[24]^. Similarly, Li *et al.* showed that artemisinin and artesunate induce ROS-dependent DNA damage and inhibit metastasis in human non-small cell lung cancer (NSCLC) A549 cells^[25]^. Clinical studies further highlight the anticancer potential of artemisinin derivatives, as artesunate has been shown to induce apoptosis in cancer cells via oxidative stress, significantly inhibiting tumor growth and improving survival in colorectal cancer (CRC) patients during Phase I trials^[8]^. Additionally, artemisinin compounds have demonstrated inhibitory effects on other malignancies, including bladder cancer (BCa), embryonal rhabdomyosarcoma (ERMS), epithelial ovarian cancer (EOC), and diffuse large B-cell lymphoma (DLBCL)^[26-30]^.

### Alantolactone compounds

**Figure 2:**
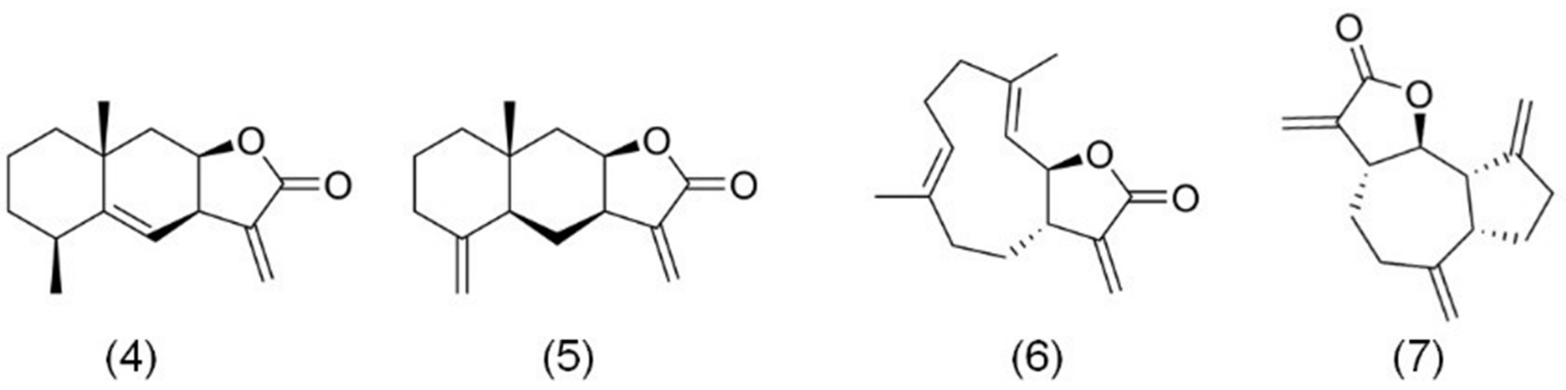
The chemical structures of alantolactone compounds. (4) Alantolactone; (5) Isoalantolactone; (6) Costunolide; (7) Dehydrocostus lactone.

Alantolactone compounds, shown in Figure 2, include alantolactone (4), isoalantolactone (5), costunolide (6), and dehydrocostus lactone (7). These sesquiterpene lactones are primarily derived from the medicinal plant *Inula helenium* L. (Asteraceae), which is known for its diverse bioactive constituents, including sesquiterpene lactones, flavonoids, amino acids, triterpenes, alkaloids, and phytosterols^[31-33]^. Among these, sesquiterpene lactones are particularly notable for their therapeutic potential. Contemporary pharmacological research has highlighted a range of biological activities for alantolactone compounds, including anticancer, anti-inflammatory, anthelmintic, antibacterial, hypoglycemic, and analgesic effects^[34]^.

Structurally, alantolactone, isoalantolactone, and dehydrocostus lactone belong to the tricyclic sesquiterpene lactone class, while costunolide features two unsaturated ring structures. Despite sharing the same molecular formula, these compounds exhibit distinct chemical arrangements that result in varied pharmacodynamics and therapeutic effects. Notably, these compounds are not exclusive to *Inula helenium* L*.*; related species, such as *Aucklandia lappa* Decne, also contain similar bioactive esquiterpene lactones^[35]^.

Recent studies have underscored the anticancer potential of alantolactone compounds. For example, Chen *et al.* reported that alantolactone suppresses the growth of esophageal adenocarcinoma (EAC) KYAE-1 cells by inducing apoptosis via inhibition of the nuclear factor E2-related factor 2 (Nrf2) pathway^[36]^. Similarly, Ren *et al.* found that alantolactone induces apoptosis in CRC HCT116 cells, effectively suppressing tumor growth^[37]^. Additionally, Yin *et al.* demonstrated the growth-inhibitory properties of alantolactone in triple-negative breast cancer (TNBC) MDA-MB-231 cells^[38]^.

Beyond alantolactone, other structurally related compounds also exhibit potent anticancer activity. Isoalantolactone, for instance, has shown significant inhibitory effects against malignant tumors such as hepatocellular carcinoma (HCC), CRC, and pancreatic cancer^[39-41]^. Costunolide has also been reported to induce apoptosis in CRC, gastric cancer (GC), and skin cancer cells^[42-44]^. Among these, dehydrocostus lactone has garnered increasing attention for its potent anticancer effects. For instance, Peng *et al.* demonstrated that dehydrocostus lactone suppresses the proliferation of EAC Eca109 cells by inducing autophagy, both *in vitro* and *in vivo*^[45]^.

### Deoxyelephantopin compounds

**Figure 3:**
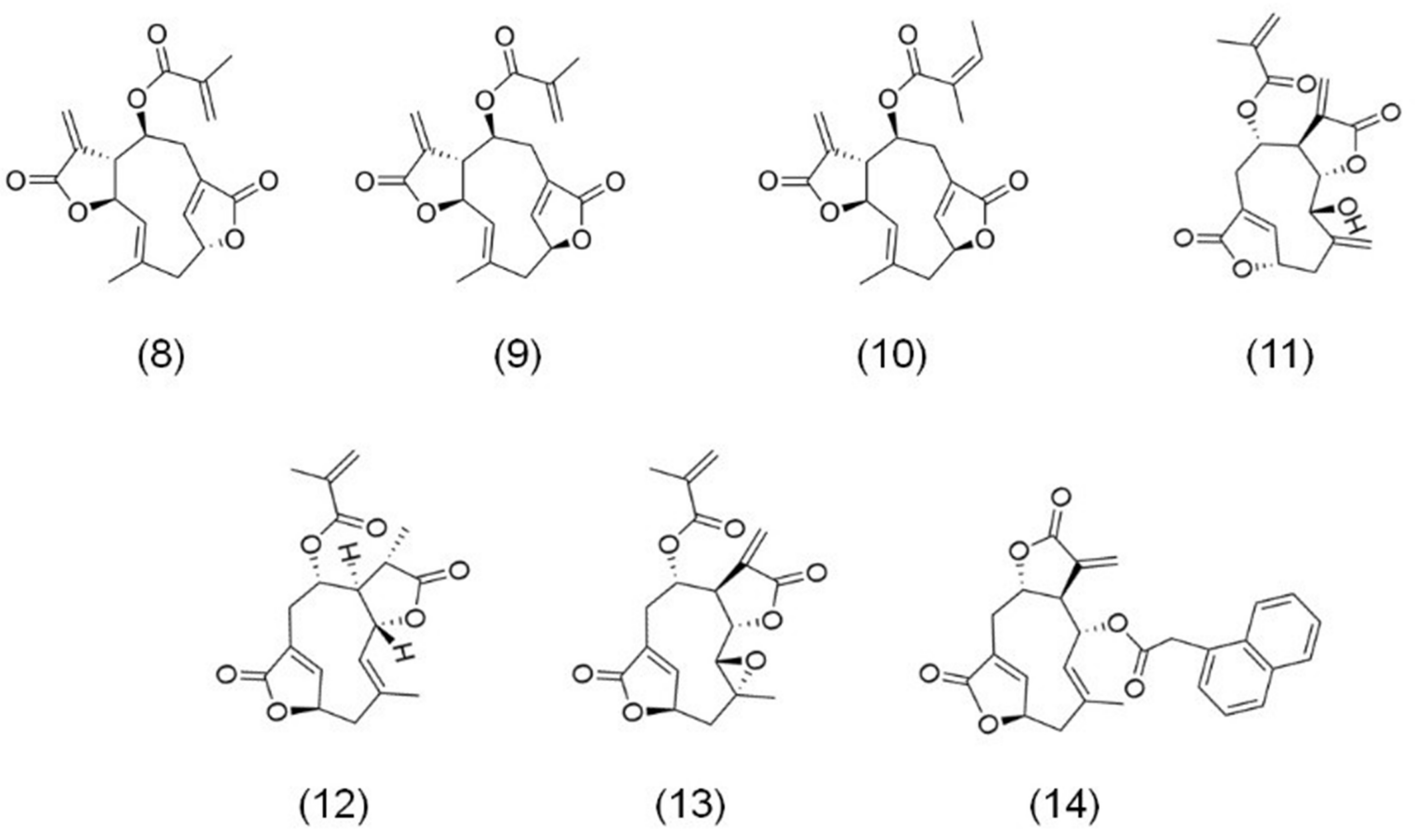
The chemical structures of deoxyelephantopin compounds. (8) Deoxyelephantopin; (9) Isodeoxyelephantopin; (10) Scabertopin; (11) Elephantopinolide A; (12) Elephantopinolide F; (13) Elephantopinolide J; and (14) DETD-35.

Deoxyelephantopin compounds, shown in Figure 3, form a subclass of sesquiterpene lactones derived from *Elephantopus scaber* L*.* These include deoxyelephantopin (8), isodeoxyelephantopin (9), scabertopin (10), elephantopinolide A (11), elephantopinolide F (12), elephantopinolide J (13), and the synthesized derivative DETD-35 (14). The discovery of deoxyelephantopin compounds dates back to the 1970s when Wolo *et al.* first isolated deoxyelephantopin from *Elephantopus* species^[46]^. Shortly thereafter, isodeoxyelephantopin, an isomer of deoxyelephantopin, was identified in the same species^[47]^. Further research leads to the identification of additional sesquiterpene lactones, including scabertopin, elephantopinolide A, elephantopinolide F, and elephantopinolide J^[48]^.

The anticancer efficacy of deoxyelephantopin compounds has been extensively studied, with several demonstrating broad-spectrum anticancer effects. For example, deoxyelephantopin itself has shown promising activity against various cancers. Kabeer *et al.* reported its efficacy against oral epidermoid cancer (KB), CRC (HCT116), and leukemia (K562) cell lines^[49]^. Similarly, its isomer, isodeoxyelephantopin, has also exhibited notable anticancer potential. Hong *et al.* found that it induces apoptosis in CRC HCT116 and RKO cells^[50]^. Building on these findings, Verma *et al.* further elucidated the mechanisms underlying isodeoxyelephantopin’s anticancer effects. They demonstrated that the compound induces G2/M phase cell cycle arrest, triggers apoptosis, and inhibits metastasis in TNBC MDA-MB-231 cells^[51]^.

In addition to deoxyelephantopin and isodeoxyelephantopin, other compounds derived from *Elephantopus scaber* L. have also shown significant anticancer activity. For instance, scabertopin has demonstrated strong anticancer effects. Gao *et al.* found that it increases ROS levels in BCa J82 and T24 cells, inducing necroptosis^[17]^. To enhance the anticancer capabilities of deoxyelephantopin compounds, researchers have explored structural modifications, leading to the synthesis of novel derivatives. One such derivative, DETD-35 (14), has shown promising results. Feng *et al.* synthesized DETD-35, which exhibits enhanced anticancer activity compared to its parent compounds^[52]^. Nakagawa-Goto *et al.* demonstrated that DETD-35 significantly inhibits the invasion and migration of TNBC MDA-MB-231 cells^[53]^. Furthermore, DETD-35 has been shown to induce apoptosis in melanoma A375 cells, cause G2/M phase cell cycle arrest, inhibit cell migration, and enhance the sensitivity of A375 cells to the BRAF inhibitor vemurafenib^[52,54]^.

Beyond deoxyelephantopin derivatives, other sesquiterpene lactones from *Elephantopus scaber* L*.* have also displayed promising anticancer effects. For example, research on elephantopinolide A and elephantopinolide F revealed their ability to induce apoptosis in glioma U87 cells, as demonstrated by Yan *et al.*^[55]^. Similarly, Bai *et al.* explored the effects of elephantopinolide J, showing that it induces G2/M phase cell cycle arrest, triggers autophagy, and promotes apoptosis in HCC HepG2 and Hep3B cells^[48]^.

### Parthenolide compounds

**Figure 4:**
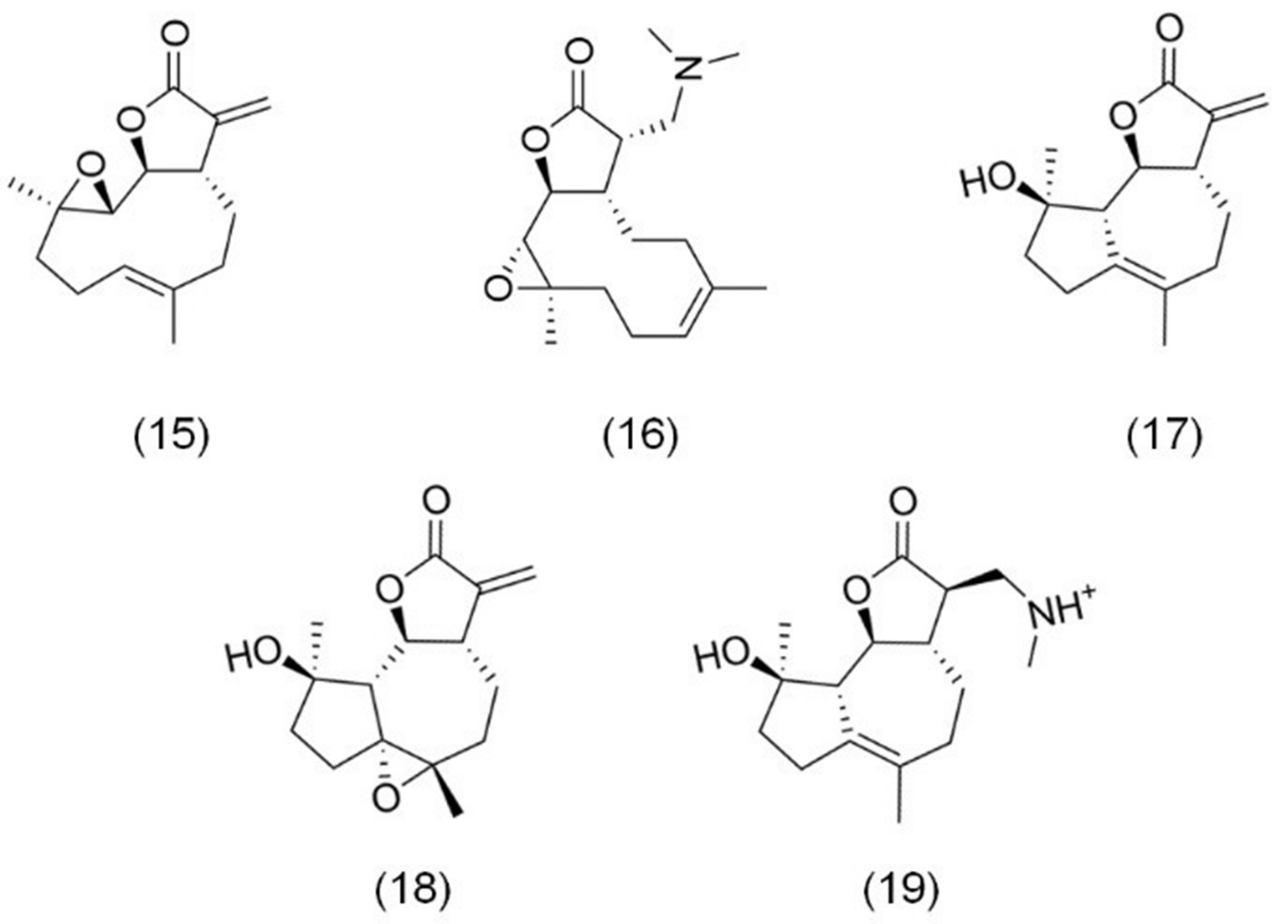
The chemical structures of parthenolide compounds. (15) Parthenolide; (16) Dimethylaminoparthenolide; (17) Micheliolide; (18) Epoxymicheliolide; (19) Dimethylaminomicheliolide.

Parthenolide compounds, a class of sesquiterpene lactones primarily from *Tanacetum parthenium* L*.*, have garnered significant attention due to their anticancer properties. This group, shown in Figure 4, includes parthenolide (15), dimethylaminoparthenolide (16), micheliolide (17), epoxymicheliolide (18), and dimethylaminomicheliolide (19).

Parthenolide, the foundational compound in this group, was initially isolated from *Tanacetum parthenium* L. and has also been found in other plant species within the Asteraceae and Magnoliaceae families. Its anticancer potential was first reported in the 1970s by Wiedhopf *et al.*, sparking widespread interest in its therapeutic applications^[56]^. However, despite its promising properties, the clinical use of parthenolide has been hindered by several challenges, including high lipophilicity, poor water solubility, and limited bioavailability^[57]^. To address these limitations, extensive research has focused on strategies to improve its pharmacokinetic profile. One effective approach has been structural modification. For example, modifying the α-methylene-γ-butyrolactone moiety via Michael addition has significantly improved water solubility while maintaining or enhancing its biological activity^[58]^. This modification led to the development of dimethylaminoparthenolide, an amino derivative with superior solubility and bioavailability compared to the parent compound^[59]^. In addition to solubility challenges, parthenolide demonstrates chemical instability under acidic and alkaline conditions, further limiting its clinical utility. To overcome this, more stable derivatives, such as micheliolide and epoxymicheliolide, have been developed. Micheliolide exhibits greater chemical stability, making it more suitable for therapeutic applications^[60]^. Its anticancer mechanism is thought to rely on its α-methylene-γ-lactone structure, which is crucial for its activity^[61,62]^. Dimethylaminomicheliolide, a Michael adduct of micheliolide, has further enhanced stability, increased anticancer activity, and shown lower toxicity compared to micheliolide itself^[63,64]^.

Extensive research has explored the anticancer activities of these sesquiterpene lactones across various cancer types. Parthenolide, for example, has demonstrated efficacy in inducing G1/G0 phase cell cycle arrest and apoptosis in several cancers. Karam *et al.* reported its ability to suppress primary effusion lymphoma (PEL) BC3 and BC1 cells, while dimethylaminoparthenolide extended the survival of NOD/SCID mice^[65]^. Similarly, Li *et al.* showed that parthenolide halts the progression of GC SGC-7901 cells in the G0/G1 phase, induces apoptosis, and inhibits cell migration and invasion, even overcoming cisplatin resistance^[66]^. Furthermore, parthenolide triggers apoptosis in NSCLC A549 and H1299 cells and significantly suppresses tumor growth in an A549 xenograft mouse model^[67]^. Additional studies have confirmed its apoptosis-inducing effects in pancreatic and cervical cancer models^[68,69]^.

Micheliolide has also shown notable anticancer effects, inducing apoptosis in GC^[70]^, glioma^[71]^, and liver cancer cells^[13]^, among others. Moreover, Zhu *et al.* demonstrated that epoxymicheliolide causes G1/G0 cell cycle arrest, triggers apoptosis, and impedes metastasis in renal cell carcinoma (RCC) 786-0 and Caki-1 cells^[62]^.

Dimethylaminomicheliolide, a key derivative of micheliolide, has exhibited remarkable anticancer properties. Zhang *et al.* reported its ability to induce apoptosis and inhibit cellular aerobic glycolysis in neuroblastoma NGP and BE2 cells, highlighting its impact on cancer metabolic reprogramming^[72]^. Additionally, studies by Yao *et al.* and Wang *et al.* revealed that dimethylaminomicheliolide effectively suppresses the migration and invasion of liver cancer cells and induces autophagy and apoptosis in glioma cells^[73,74]^.

### Sesquiterpene lactones derived from *Centipeda minima*

**Figure 5:**
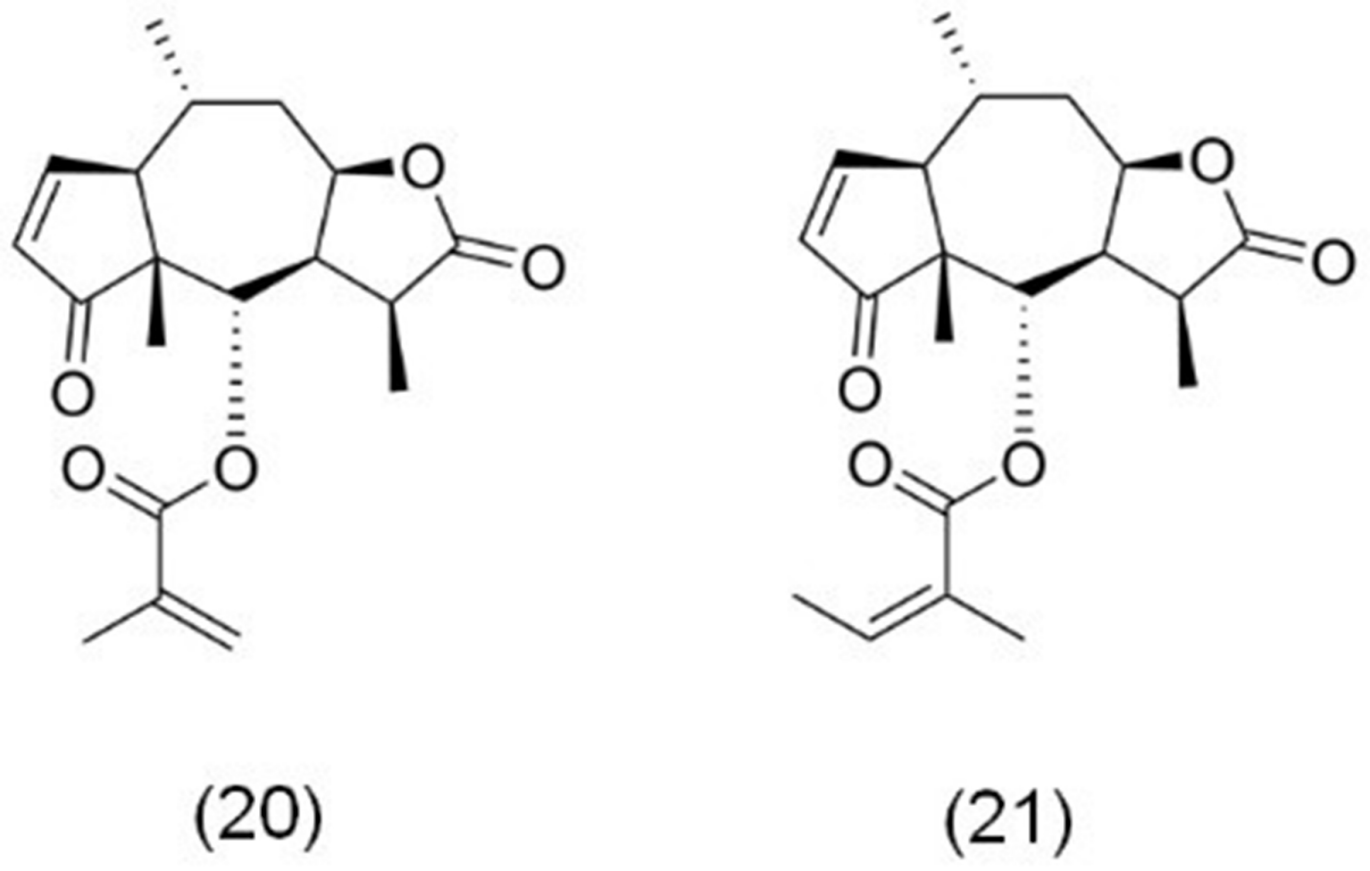
The chemical structures of sesquiterpene lactones derived from *Centipeda minima.* (20) Arnicolide D; (21) Brevilin A.

In recent years, several sesquiterpene lactones have been identified in *Centipeda minima* L., with arnicolide D (20) and brevilin A (21) emerging as the most prominent compounds for their anticancer activity, as shown in Figure 5. The structural configuration of these compounds is notably distinct from those derived from other plant species, particularly due to the presence of an α,β-unsaturated lactone moiety within the A ring. This moiety functions as a Michael acceptor, significantly enhancing their biological activity^[75]^. Furthermore, the C/B ring junction at positions 7 and 8, along with diverse substitutions at position 6 (e.g., angeloyl, methacryloyl, and methylcrotonyl groups), highlights their potential specificity in biological interactions^[76]^. These unique chemical features establish arnicolide D and brevilin A as promising candidates for the development of innovative anticancer agents.

Our recent studies have highlighted the potent anticancer effects of arnicolide D across various cancers. Arnicolide D consistently induces apoptosis and G2/M phase cell cycle arrest, as observed in nasopharyngeal carcinoma (NPC) CNE-2 cells^[77]^, TNBC MDA-MB-231 cells^[78]^, and melanoma B16F10 cells^[79]^. Additionally, our latest research revealed that arnicolide D induces oncosis in HCC cells through ER stress mediated by CHOP and ATF4 pathways^[80]^. Other studies have further demonstrated arnicolide D’s ability to suppress proliferation and induce apoptosis in osteosarcoma (OS) cells^[81]^.

Beyond arnicolide D, our group has also explored the anticancer activity of brevilin A in various cancer models. Brevilin A demonstrates strong anti-melanoma effects by inducing apoptosis and inhibiting metastasis *in vitro*, as well as significantly suppressing tumor growth in a xenograft A375 mouse mode^[82]^. It also induces G2/M phase arrest and apoptosis in NPC CNE-2 cells, further supporting its potential against NPC^[83]^. Additionally, Saleem *et al.* reported that brevilin A triggers apoptosis and ER stress while inducing G2/M phase cell cycle arrest. Furthermore, brevilin A has been shown to inhibit the migration and invasion of MCF-7 breast cancer cells^[84]^. You *et al.* also demonstrated that brevilin A promotes both apoptosis and autophagy in CRC CT26 cells, underscoring its broad-spectrum anticancer efficacy^[85]^.

### Sesquiterpene lactones from other sources

**Figure 6:**
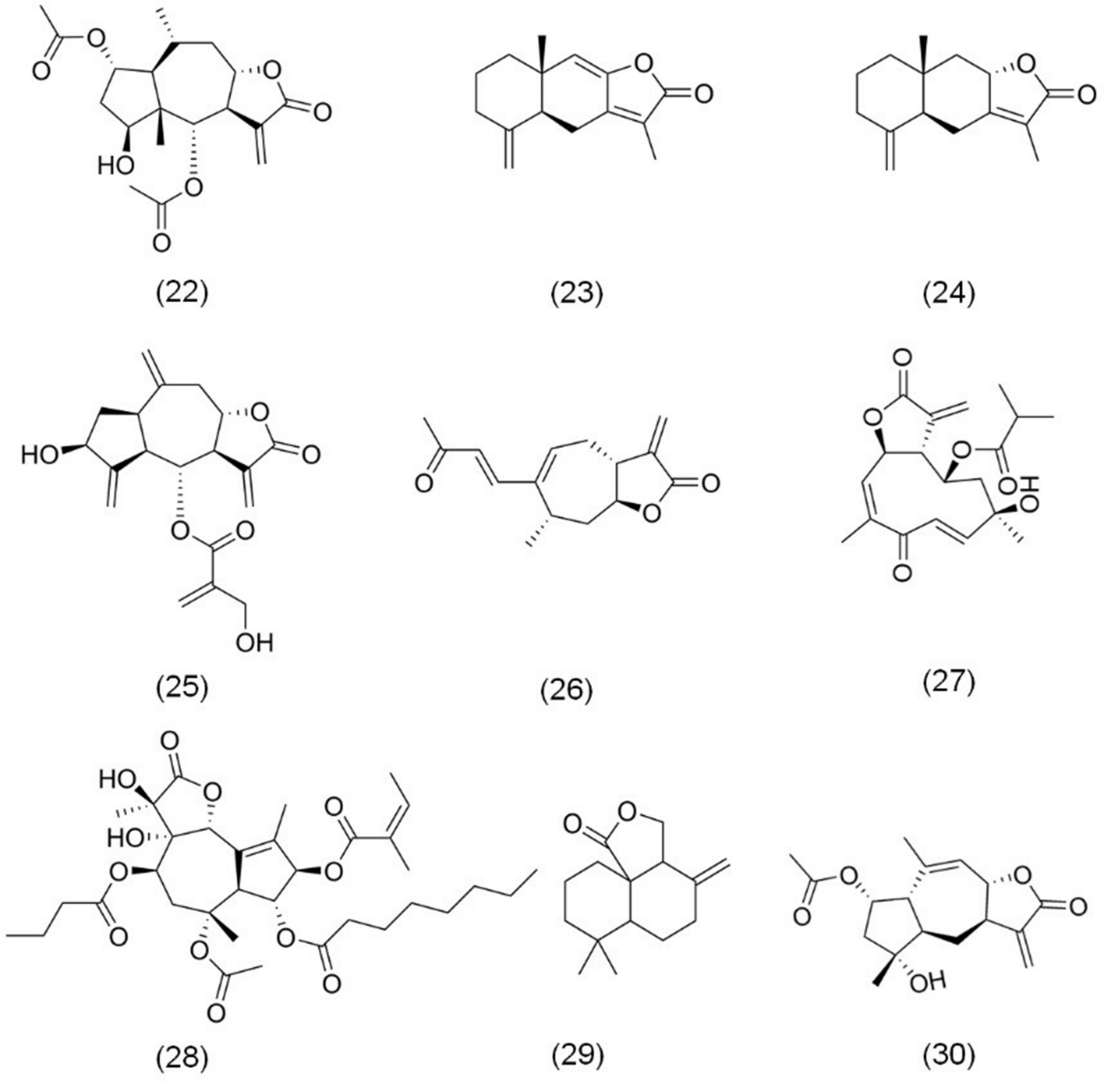
The chemical structures of sesquiterpene lactones from other sources. (22) Britannin; (23) Atractylenolide I; (24) Atractylenolide II; (25) Hemistepsin A; (26) Xanthatin; (27) Tagitinin C; (28) Thapsigargin; (29) Antrocin; (30) Gaillardin.

Recent studies have identified numerous natural sesquiterpene lactones with significant anticancer activity from various plant sources, as shown in Figure 6. Among these, britannin (22), a sesquiterpene lactone originally derived from *Inula aucheriana* Thunb. of the Asteraceae family, has demonstrated notable anticancer properties in various cancer models^[86]^. Beyond its initial identification, britannin has also been extracted from other *Inula* species, including *Inula japonica* and *Inula lineariifolia* Turcz^[87]^. Studies have revealed that britannin inhibits both proliferation and angiogenesis in CRC HCT116 cells, highlighting its potential as an anticancer strategy^[88]^. In HCC models, britannin triggers apoptosis through both extrinsic and intrinsic pathways and promotes autophagy, effectively suppressing cancer initiation and progression^[27]^.

Two additional sesquiterpene lactones, atractylenolide I (23) and atractylenolide II (24), have been isolated from *Atractylodes macrocephala* Koidz., and exhibit potent anticancer activities across multiple cancer cell lines^[89]^. Atractylenolide I induces apoptosis and significantly hinders invasiveness in CRC COLO205 and HCT116 cells. In an *in vivo* mouse xenograft model on CRC, atractylenolide I effectively inhibits tumor growth^[90]^. Meanwhile, atractylenolide II induces apoptosis and G2/M phase cell cycle arrest in human prostate cancer (PCa) cells (DU145 and LNCaP), thereby suppressing cell proliferation^[91]^.

Hemistepsin A (25), derived from *Hemistepta lyrata* Bunge, represents another promising sesquiterpene lactone for cancer therapy^[92]^. It exerts potent anticancer effects in malignancies such as PCa and HCC by inducing autophagy and apoptosis, as well as inhibiting cell cycle progression^[15,93]^.

Another group of sesquiterpene lactones can be extracted from *Xanthium strumarium* L*.*^[94]^. Among these, xanthatin (26), a bicyclic sesquiterpene lactone, features a highly reactive α-methylene-γ-butenolide moiety critical for its pharmacological effects^[95]^. Shi *et al.* demonstrated that xanthatin inhibits the viability of HCC cells (SMMC-7721, Bel-7402, and HepG2) by inducing G2/M phase arrest, apoptosis, and ER stress^[96]^.

Tagitinin C (27), initially extracted from *Tithonia diversifolia* A. Gray, is another notable sesquiterpene lactone with potent anticancer activity^[97]^. Research conducted by Wei *et al.* revealed that tagitinin C induces ER stress, ferroptosis, and G2/M cell cycle arrest in CRC HCT116 cells, contributing to its anticancer properties^[98]^.

Thapsigargin (28), derived from *Thapsia garganica* L., stands out due to its unique trans-C7α-C6β-γ lactone ring configuration, which includes trans-hydroxyl groups at positions C7 and C11^[99]^. This structural uniqueness underpins its potent biological activity. Huang *et al.* demonstrated that thapsigargin induces apoptosis and inhibits proliferation in PCa PC3 cells^[100]^. Additionally, Wu *et al.* showed that thapsigargin induces ER stress and apoptosis in adrenocortical carcinoma (ACC) SW-13 cells, while suppressing cell migration and invasion^[101]^.

Antrocin (29), a cyclic sesquiterpene lactone first isolated from the fungus *Antrodia cinnamomea* by Chiang *et al.* in 1995, has shown significant potential as a novel anticancer agent^[102]^. Chen *et al.* reported that antrocin markedly reduces tumorigenicity and stemness in TNBC both *in vitro* and *in vivo*^[102]^. Furthermore, Chiu *et al.* found that antrocin induces apoptosis and reduces metastasis in BCa 5637 cells, underscoring its therapeutic potential for aggressive cancers^[103]^.

Gaillardin (30), a pseudoguaianolide-type sesquiterpene lactone derived from *Inula oculus-christi*, another Asteraceae species, has also garnered attention for its anticancer properties^[104]^. Roozbehani *et al.* discovered that gaillardin impedes the proliferation of GC cells (AGS and MKN45) by inducing apoptosis^[104]^.

## SIGNAL PATHWAYS INVOLVED IN THE CANCER DRUG RESISTANCE-OVERCOMING EFFECT OF SESQUITERPENE LACTONES

Signal transduction pathways are crucial in cancer progression, regulating key cellular processes like proliferation, survival, and drug resistance. This section focuses on five major pathways involved in cancer progression and drug resistance, specifically examining how sesquiterpene lactones modulate these pathways to enhance anticancer effects and overcome resistance mechanisms.

### PI3K/Akt/mTOR pathway

**Figure 7:**
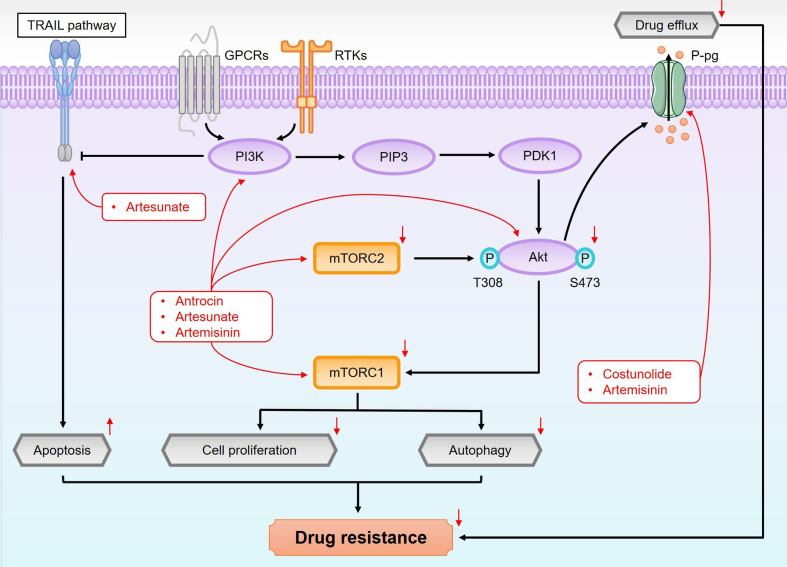
An overview of the PI3K/Akt/mTOR pathway and its role in overcoming drug resistance mediated by sesquiterpene lactones. This pathway is activated by cell surface receptors, including RTKs and GPCRs, leading to PI3K activation and the generation of PIP3. PIP3 facilitates the phosphorylation of Akt and promotes the membrane translocation of both Akt and PDK1, triggering downstream signaling through mTOR. This activation enhances drug resistance by upregulating ABC transporter expression, thereby increasing drug efflux. Moreover, the PI3K/Akt/mTOR pathway regulates key processes such as autophagy and tumor proliferation through mTOR modulation. Its interaction with the TRAIL pathway further amplifies the complexity of drug resistance networks by influencing apoptosis, reinforcing the role of this pathway in resistance mechanisms. PI3K: Phosphoinositide 3-kinase; RTKs: receptor tyrosine kinases; GPCRs: G protein-coupled receptors; PIP3: phosphatidylinositol 3,4,5-triphosphate; PDK1: phosphoinositide-dependent kinase-1; ABC: ATP-binding cassette; TRAIL: tumor necrosis factor-related apoptosis-inducing ligand.

The PI3K/serine-threonine protein kinase Akt (also known as protein kinase B, PKB)/mammalian target of rapamycin (mTOR) pathway, shown in Figure 7, is critical for regulating various aspects of cancer biology, such as cell survival, growth, metabolism, and drug resistance^[105,106]^. Dysregulation of this pathway, often initiated by cell surface receptors such as receptor tyrosine kinases (RTKs) and G protein-coupled receptors (GPCRs), is a hallmark of many cancers and a major contributor to therapeutic resistance, making it a key target for intervention. PI3K is a family of lipid kinases, classified into three classes, with Class I being the most strongly implicated in tumorigenesis through the generation of phosphatidylinositol 3,4,5-triphosphate (PIP3)^[107]^. The accumulation of PIP3 facilitates the membrane translocation of phosphoinositide-dependent kinase-1 (PDK1) and Akt^[108,109]^, leading to the phosphorylation of Akt at threonine 308 and serine 473, thereby activating downstream signaling pathways, including mTOR^[110]^. mTOR exists in two distinct complexes: mTORC1, which regulates cellular growth, protein synthesis, and cell cycle progression, and mTORC2, which primarily governs cytoskeletal organization and cell survival^[111,112]^.

This pathway is not only for cancer cell proliferation but also plays a crucial role in developing resistance to various chemotherapeutic agents. Increased expression and activation of PI3K and Akt in multiple tumor types lead to enhanced mTOR activity, which promotes tumor initiation, progression, and metastasis^[113]^. This pathway contributes to drug resistance by inhibiting apoptotic signaling and promoting cell survival under stress conditions, thus complicating treatment efficacy^[114]^. Studies have shown that sesquiterpene lactones like antrocin and artesunate target key components of this pathway. These compounds inhibit PI3K and Akt activation, downregulate mTOR signaling, and reverse drug resistance in HCC cells, improving therapeutic outcomes^[115,116]^. Additionally, artemisinin has been shown to enhance cancer cell sensitivity to 5-fluorouracil (5-FU) by inhibiting Akt phosphorylation, further supporting the potential of sesquiterpene lactones in overcoming drug resistance^[117]^.

The PI3K/Akt/mTOR pathway is also crucial in MDR, a significant challenge in cancer therapy^[118,119]^. Activation of this pathway upregulates the expression and activity of ATP-binding cassette (ABC) transporters, such as P-glycoprotein (P-gp), which expels chemotherapeutic agents from the cytoplasm, lowering intracellular drug concentrations and contributing to MDR^[120]^. Sesquiterpene lactones can reverse MDR by downregulating P-gp expression and restoring chemotherapeutic efficacy. For example, costunolide enhances the sensitivity of K562/ADR chronic myeloid leukemia (CML) cells to doxorubicin through P-gp downregulation^[121]^. Similarly, artemisinin increases the sensitivity of cisplatin-resistant GC cells (SGC7901/DDP) to cisplatin by inhibiting P-gp activity^[122]^.

Resistance to tumor necrosis factor-related apoptosis-inducing ligand (TRAIL) is also often mediated by the PI3K/Akt/mTOR pathway^[123]^. TRAIL, a member of the tumor necrosis factor (TNF) family, induces apoptosis by binding to its receptors, yet many cancer cells exhibit resistance to TRAIL-induced cell death, which limits its therapeutic potential^[124]^. The pathway modulates TRAIL-induced apoptosis by phosphorylating and activating downstream signaling molecules and regulating TRAIL receptor expression, which diminishes TRAIL’s effectiveness. Inhibiting the PI3K/Akt pathway enhances TRAIL-induced apoptosis by restoring receptor expression and promoting apoptosis in cancer cells. For instance, artesunate has been demonstrated to enhance TRAIL-induced apoptosis in human cervical cancer cells, overcoming TRAIL resistance^[125]^. These findings suggest that sesquiterpene lactones, by targeting critical nodes in the PI3K/Akt/mTOR pathway, hold promise as strategies for enhancing chemotherapy efficacy and overcoming drug resistance in various cancers.

### NF-κB pathway

**Figure 8:**
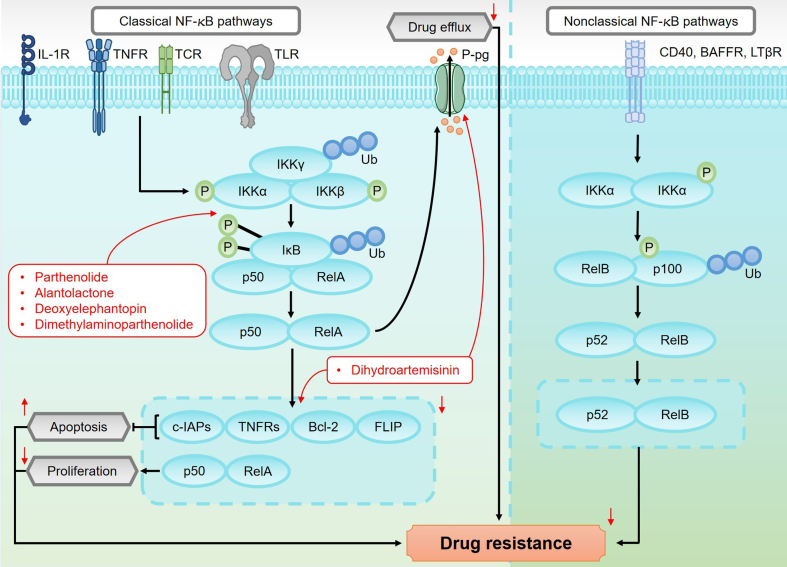
An overview of the canonical and non-canonical NF-κB pathways and their roles in regulating drug resistance in sesquiterpene lactones. The canonical NF-κB pathway is activated by pro-inflammatory cytokines and stress signals, which trigger the phosphorylation of IKKs. This phosphorylation leads to the degradation of IκB proteins, releasing NF-κB dimers, such as p50/p65. These dimers then translocate to the nucleus, where they regulate the expression of genes involved in inflammation, immune response, and tumor progression. In contrast, the non-canonical NF-κB pathway is activated by specific members of the TNF receptor superfamily, such as BAFFR, CD40, and LTβR. This activation leads to the activation of NIK, which in turn activates IKKα. IKKα phosphorylates p100, resulting in its partial degradation to generate p52. The p52/RelB complex then translocates to the nucleus, where it regulates genes associated with immunity and tumorigenesis. Activation of the canonical NF-κB pathway promotes the upregulation of P-gp, which enhances drug efflux and contributes to drug resistance. Additionally, NF-κB activation increases the expression of anti-apoptotic proteins such as FLIP, c-IAPs, TNFRs, and Bcl-2. These proteins inhibit apoptosis, thereby promoting chemotherapy resistance. NF-κB: Nuclear factor kappa-light-chain-enhancer of activated B cells; IKKs: IκB kinases; TNF: tumor necrosis factor; NIK: NF-κB-inducing kinase; P-gp: P-glycoprotein; FLIP: FLICE-inhibitory protein; TNFRs: tumor necrosis factor receptors.

The NF-κB pathway plays a crucial role in regulating inflammation, immune response, and cancer development^[126,127]^. As shown in Figure 8, this pathway consists of five key members: RelA (p65), RelB, and c-Rel, NF-κB1 (p50), NF-κB2 (p52). In quiescent cells, NF-κB proteins are sequestered in the cytoplasm by inhibitors from the IκB family, which prevent their activation^[128]^. The NF-κB pathway is activated through two distinct signaling routes: the canonical (classical) pathway and the non-canonical (alternative) pathway.

The canonical NF-κB pathway is characterized by rapid, transient activation in response to pro-inflammatory cytokines, microbial components, and stress signals. It is primarily initiated by key receptors such as interleukin-1 receptors (IL-1Rs), Toll-like receptors (TLRs), T-cell receptors (TCRs), and tumor necrosis factor receptors (TNFRs). Upon activation, IκB kinases (IKKs) are phosphorylated, leading to the ubiquitin-dependent degradation of IκB proteins. This degradation liberates NF-κB dimers (typically p50/p65), allowing their translocation into the nucleus to regulate the expression of target genes^[129]^. In contrast, the non-canonical NF-κB pathway features slower and more sustained activation. It is primarily activated by specific members of the TNF receptor superfamily, such as BAFFR, CD40, and LTβR. Activation of NF-κB-inducing kinase (NIK) results in the phosphorylation of IKKα, which subsequently phosphorylates p100. This phosphorylation leads to the partial degradation of p100 to p52. The p52/RelB complex then translocates to the nucleus, where it regulates genes involved in immune modulation and tumor progression^[130]^.

Dysregulated activation of the canonical NF-κB pathway is implicated in several pathological conditions, including tumorigenesis and the development of drug resistance. Abnormal upstream signaling, excessive phosphorylation or degradation of IκB proteins, and persistent or overactive IKK complex expression can result in sustained NF-κB activation. This prolonged activation promotes tumor initiation, progression, and resistance to therapy^[131]^. Sesquiterpene lactones exert their antitumor effects by inhibiting IKK activity, blocking IκB phosphorylation and degradation, and preventing the NF-κB/p65 dimer from translocating into the nucleus. As a result, NF-κB nuclear accumulation is reduced, and its transcriptional activity is diminished, enhancing tumor cell sensitivity to chemotherapy. For instance, alantolactone inhibits TNF-α-induced IκBα phosphorylation and blocks NF-κB/p65 nuclear translocation, inducing apoptosis in imatinib-resistant CML cells and overcoming their resistance to imatinib^[132]^. Similarly, parthenolide enhances sensitivity to oxaliplatin by suppressing NF-κB/p65 signaling in lung cancer cells, mitigating oxaliplatin resistance^[133]^. Resistance to gemcitabine, a standard chemotherapy for pancreatic cancer, is closely associated with aberrant NF-κB activation^[134]^. Studies show that both deoxyelephantopin and dimethylaminoparthenolide, when combined with gemcitabine, reverse resistance in pancreatic cancer cells by inhibiting NF-κB/p65 signaling. This combined treatment approach significantly enhances gemcitabine’s therapeutic efficacy in clinical settings^[135,136]^.

The NF-κB pathway is also a key driver of MDR. Upon activation, the NF-κB/p65 dimer translocates to the nucleus and upregulates the expression of the gene encoding P-gp. P-gp is involved in actively expelling chemotherapeutic agents from cancer cells, reducing intracellular drug concentrations and diminishing therapeutic efficacy^[137]^. By inhibiting the NF-κB pathway, sesquiterpene lactones have been shown to enhance chemosensitivity by downregulating P-gp expression. For example, dihydroartemisinin improves the sensitivity of HCC cells to doxorubicin via this mechanism^[138]^.

In addition to facilitating drug efflux, NF-κB activation contributes to resistance by upregulating various anti-apoptotic proteins, such as FLICE-inhibitory protein (FLIP), c-IAPs, TNFRs, and Bcl-2^[139]^. These proteins enhance tumor cell survival and confer resistance to chemotherapy-induced apoptosis, thereby contributing to drug resistance^[140,141]^. Sesquiterpene lactones can inhibit NF-κB activity, leading to significant downregulation of these anti-apoptotic proteins and promoting tumor cell apoptosis. For example, dihydroartemisinin has been shown to overcome gemcitabine resistance in pancreatic cancer cells both *in vitro* and *in vivo*, enhancing therapeutic efficacy^[138]^. These findings highlight the potential of sesquiterpene lactones in inhibiting NF-κB signaling, overcoming chemotherapy resistance, and improving treatment outcomes.

### Wnt/β-catenin pathway

The Wnt signaling pathway regulates a wide range of cellular processes, including tumor cell growth, differentiation, apoptosis, and autophagy. This pathway plays a critical role in tumorigenesis and has become an important target for therapeutic interventions^[142]^. Conserved throughout evolution, the Wnt/β-catenin pathway is essential for numerous physiological functions^[143]^, and its dysregulation is associated with the initiation and progression of various malignancies^[82,144,145]^.

**Figure 9:**
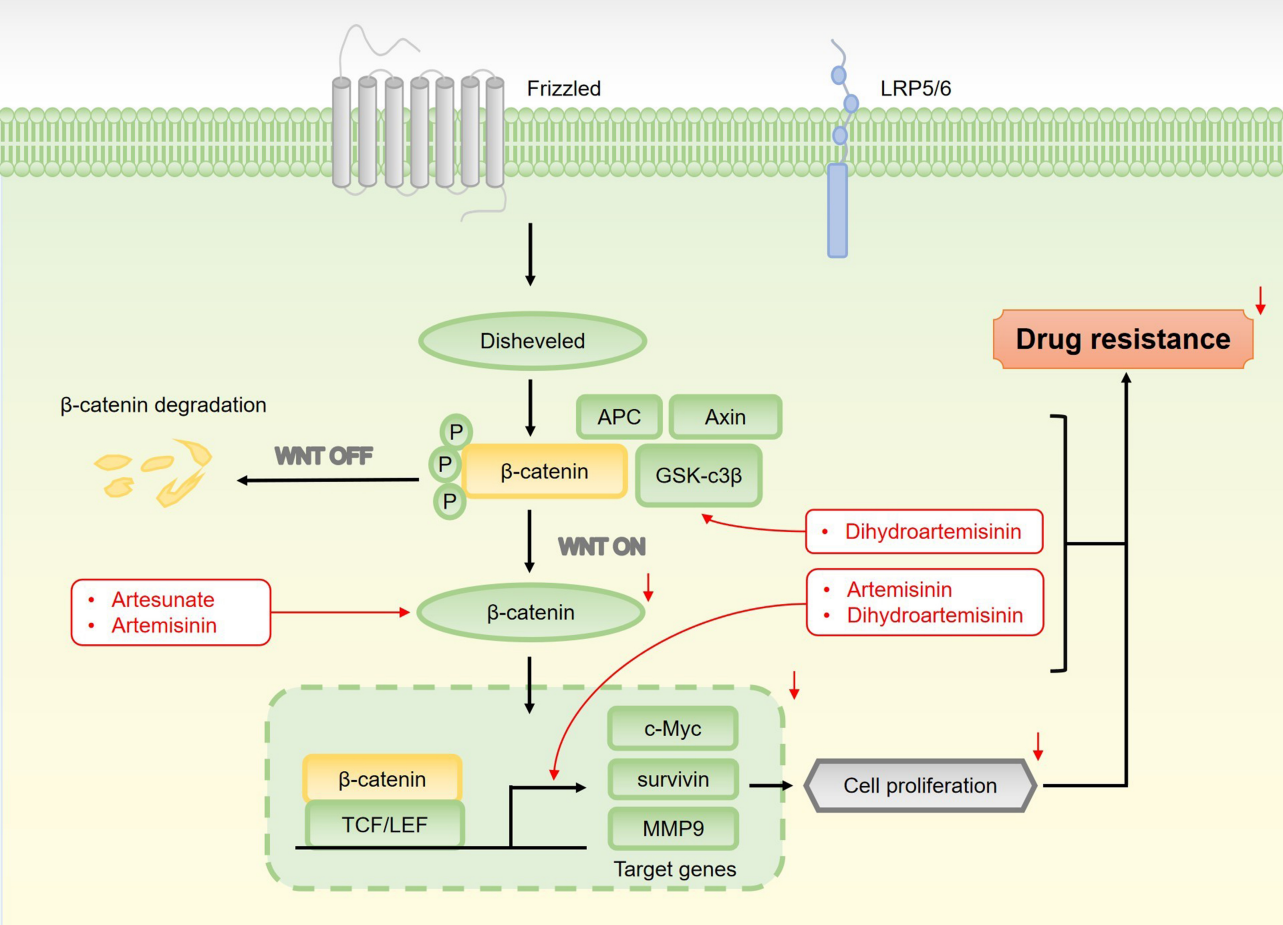
An overview of the Wnt/β-catenin pathway and its critical role in mediating drug resistance of sesquiterpene lactones in cancer cells. The pathway is activated when Wnt ligands bind to the FZD and LRP-5/6 receptor complex on the cell surface, triggering downstream signaling events. In the absence of Wnt ligands, β-catenin is ubiquitinated and degraded by a destruction complex consisting of Axin, APC, and GSK3β. However, when the Wnt pathway is activated, this destruction complex is inhibited, preventing β-catenin degradation. Consequently, β-catenin accumulates in the cytoplasm and translocates to the nucleus, where it activates the transcription of target genes. By activating β-catenin, the Wnt pathway enhances drug resistance through the upregulation of genes such as c-MYC, survivin, and MMP9, which play crucial roles in promoting cell proliferation. Sesquiterpene lactones counteract drug resistance by modulating β-catenin, GSK-3β, and the expression of downstream genes. FZD: Frizzled; LRP-5/6: lipoprotein receptor-related protein 5/6; APC: adenomatous polyposis coli; GSK3β: glycogen synthase kinase 3 beta.

As shown in Figure 9, β-catenin is a central mediator in this signaling cascade, acting both as a cell adhesion protein and as a key effector in Wnt signaling^[146]^. The pathway is initiated when Wnt ligands bind to a receptor complex on the cellular membrane, consisting of low-density lipoprotein receptor-related protein 5/6 (LRP-5/6) and Frizzled (FZD). In the absence of Wnt ligands, β-catenin is ubiquitinated and degraded by a destruction complex, which includes Axin, adenomatous polyposis coli (APC), and glycogen synthase kinase 3 beta (GSK3β). Upon Wnt ligand binding, the destruction complex is inhibited, leading to the accumulation and nuclear translocation of β-catenin. In the nucleus, β-catenin interacts with T-cell factor/lymphoid enhancer-binding factor (TCF/LEF) transcription factors and co-activators such as Pygopus and Bcl-9, activating target gene transcription^[147]^. In addition to these roles, β-catenin also intersects with autophagy pathways, either by activating the PI3K/Akt/mTOR pathway or promoting the fusion of autophagosomes with lysosomes^[101,111,148,149]^.

While the Wnt/β-catenin is crucial for embryonic development and tissue homeostasis, its aberrant activation is commonly observed in many cancers, making it a prime target for anticancer therapies. Notably, this pathway is also involved in cancer drug resistance. Under normal conditions, Wnt/β-catenin signaling is tightly regulated through β-catenin stability. However, in various malignancies, abnormal accumulation of β-catenin leads to persistent activation of Wnt/β-catenin signaling, driving tumor progression and contributing to drug resistance^[150-152]^.

Regulating β-catenin expression is therefore critical for how sesquiterpene lactones modulate Wnt/β-catenin signaling to combat drug resistance. In glioblastoma, the chemotherapeutic agent temozolomide (TMZ), a DNA methylator, faces resistance due to Wnt/β-catenin activation. This pathway enhances the expression of MGMT (O6-methylguanine-DNA-methyltransferase), contributing to TMZ resistance. Artesunate counteracts this by downregulating β-catenin expression, inhibiting Wnt/β-catenin activation, and suppressing MGMT, thereby reversing TMZ resistance^[153]^. Similarly, in EC, Wnt/β-catenin signaling plays a critical role in drug resistance. Overactivation of β-catenin promotes cell survival and proliferation, reducing the effectiveness of oxaliplatin. Artemisinin enhances the sensitivity of EC cells to oxaliplatin by inhibiting Wnt/β-catenin signaling^[154]^.

One effective strategy to reduce β-catenin levels is to enhance its degradation. GSK-3β, a negative regulator of the Wnt/β-catenin pathway, phosphorylates β-catenin, leading to its degradation^[155]^. Sesquiterpene lactones activate GSK-3β, promoting β-catenin phosphorylation and degradation. This inhibits Wnt/β-catenin signaling and mitigates drug resistance. For example, dihydroartemisinin targets GSK-3β to suppress Wnt/β-catenin signaling in colon cancer, inhibiting tumor progression and overcoming resistance to capecitabine^[156]^.

In the Wnt/β-catenin pathway, β-catenin binds to transcription factors such as TCF/LEF to activate downstream gene expression. Reducing β-catenin expression limits its interaction with these factors, disrupting the pathway’s support of cell proliferation and drug resistance^[157]^. Sesquiterpene lactones can indirectly inhibit these transcription factors by reducing β-catenin accumulation in the nucleus, enhancing chemotherapy sensitivity. For instance, dihydroartemisinin targets the Wnt/β-catenin/TCF7/MMP9 pathway, synergizing with capecitabine to enhance anticancer effects and overcome drug resistance in CRC^[156]^. In EC, upregulation of Wnt/β-catenin target genes, such as c-Myc and survivin, promotes tumor progression and resistance. Artemisinin significantly reduces c-Myc and survivin expression, induces apoptosis, and reverses oxaliplatin resistance by inhibiting Wnt/β-catenin signaling^[154]^. Thus, sesquiterpene lactones offer promising therapeutic strategies by inhibiting the overactivation of the Wnt/β-catenin pathway, demonstrating potent anti-resistance effects.

### MAPK/ERK pathway

**Figure 10:**
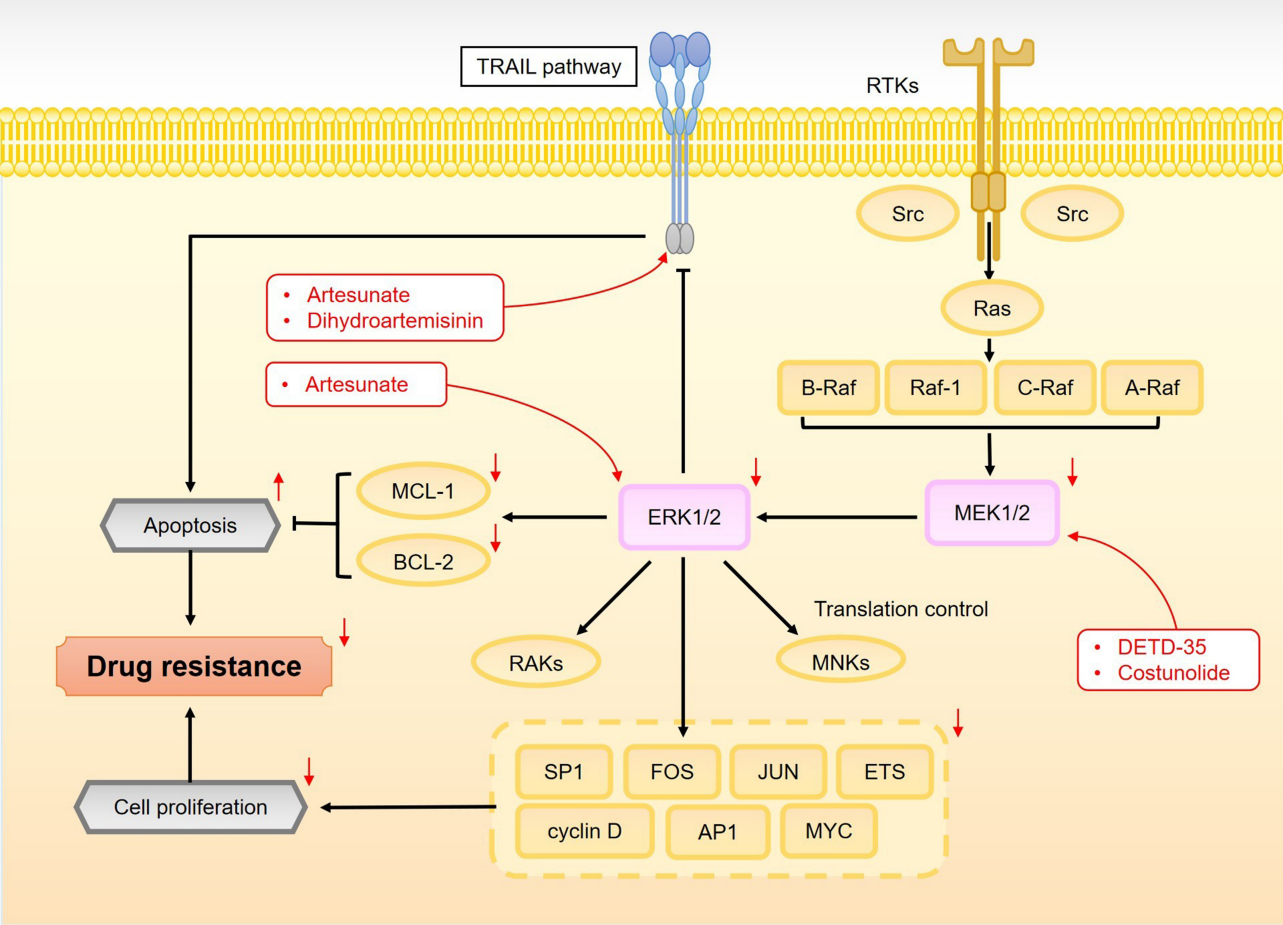
An overview of the MAPK/ERK pathway and its role in overcoming cancer cell drug resistance by sesquiterpene lactones. The MAPK/ERK pathway is a critical signaling cascade involving three classes of kinases: MAPKKK, MAPKK, and MAPK, which sequentially phosphorylate downstream targets. It begins with the activation of MAPKKKs, which subsequently activate MEK1 and MEK2. These, in turn, lead to the activation of ERK1 and ERK2, the final effectors of the cascade. Alterations in components of the Ras/BRAF/MEK/ERK pathway can result in the sustained activation of downstream signaling. This continuous activation promotes the expression of genes involved in cell survival and proliferation, enhances anti-apoptotic signaling, and ultimately contributes to chemotherapy resistance. Furthermore, the MAPK/ERK pathway interacts with the TRAIL pathway, working together to regulate drug resistance. MAPK: Mitogen-activated protein kinase; ERK: extracellular signal-regulated kinase; MAPKKK: MAPK kinase kinase; MAPKK/MEK: MAPK kinase; TRAIL: tumor necrosis factor-related apoptosis-inducing ligand.

The MAPK/ERK pathway is a crucial signaling cascade that regulates key cellular processes, including proliferation, differentiation, apoptosis, and inflammatory responses^[158,159]^. As shown in Figure 10, this pathway involves three primary classes of kinases: MAPK kinase kinase (MAPKKK), MAPK kinase (MAPKK/MEK), and MAPK, which sequentially phosphorylate downstream targets, thereby propagating the signal^[160]^. In the canonical MAPK/ERK pathway, MAPKKKs, such as A-RAF, B-RAF, and RAF-1 (C-RAF), activate the MAPKKs MEK1 and MEK2, which, in turn, activate the final effectors, ERK1 and ERK2^[161]^. ERK1/2 are central to translating extracellular signals into intracellular responses, and four major MAPK cascades have been identified: ERK1/2, c-Jun N-terminal kinase (JNK), p38 MAPK, and ERK5^[161]^. ERK1/2 activation occurs through phosphorylation by MEK1/2, which are activated by serine/threonine kinases from the Raf family^[162]^. Once phosphorylated, ERK1/2 modulate transcription factors, such as ELK1, ETS, FOS, JUN, MYC, and SP1, to regulate gene expression related to cellular proliferation and cell cycle control^[163]^. Additionally, ERK1/2 phosphorylate intracellular kinases, including RAKs, MNKs, and MSKs, which contribute to cellular growth and attachment^[164]^. Under normal physiological conditions, the MAPK/ERK pathway plays a tumor-suppressive role by promoting cellular senescence and facilitating protein degradation, both essential for cell cycle progression and survival^[165]^. However, dysregulation of this pathway, often dirven by abnormal activation of RTKs and their downstream effectors, such as RAS, PI3K, and SRC, leads to its upregulation, thereby driving cancer progression and drug resistance.

The MAPK/ERK pathway’s pivotal role in cancer initiation, progression, and resistance makes it a key target for therapeutic intervention. In many cancers, excessive activation of this pathway, driven by molecules like Ras, B-Raf, and MEK, promotes cell proliferation and survival, contributing to drug resistance^[166-168]^. Sesquiterpene lactones have emerged as potential inhibitors of this pathway, reducing the activity of key molecules within the cascade and suppressing both cancer cell proliferation and resistance. For instance, osimertinib, which is effective in treating NSCLC, faces limitations due to acquired resistance. Costunolide has been shown to directly inhibit MEK1/2, thereby blocking MAPK/ERK signaling and reversing tumor cell resistance to osimertinib^[169]^. Similarly, vemurafenib, a BRAF (V600E)-specific inhibitor for melanoma, encounters resistance, which can be overcome by combining it with DETD-35. This combination suppresses MEK expression, inhibits MAPK/ERK signaling, and counteracts vemurafenib resistance^[52]^.

Phosphorylation of ERK1/2 is a critical event in the activation of the MAPK/ERK pathway. Abnormal activation of ERK1/2 accelerates tumor progression and induces drug resistance^[170]^. In this context, sesquiterpene lactones inhibit ERK1/2 phosphorylation, disrupt downstream signaling, and reduce resistance to chemotherapy. As a result, combination therapies targeting ERK1/2-related resistance have gained increasing attention in research. In ovarian cancer, artesunate and methotrexate conjugates inhibit ERK1/2 phosphorylation, suppress MAPK/ERK signaling, and enhance drug sensitivity^[171]^. In HCC, continuous ERK1/2 activation during cisplatin treatment promotes cell survival and inhibits cisplatin-induced apoptosis, contributing to resistance. However, the combination of dihydroartemisinin with cisplatin reduces p-ERK/ERK expression and reverses cisplatin resistance^[172]^.

In addition to its role in chemotherapy resistance, aberrant MAPK/ERK activation is a significant factor in TRAIL resistance. Many tumor cells fail to undergo apoptosis following TRAIL treatment due to persistent MAPK/ERK activation^[173]^. Modulating MAPK/ERK activity has been shown to enhance TRAIL-induced apoptosis, thus overcoming resistance. Sesquiterpene lactones have emerged as effective TRAIL sensitizers, potentiating TRAIL’s anticancer effects in resistant tumors. For example, artesunate reduces MAPK/ERK overactivation and decreases anti-apoptotic factors, such as Bcl-2, thus enhancing TRAIL-induced apoptosis and reversing TRAIL resistance in human cervical cancer cells^[125]^. These findings highlight the potential of sesquiterpene lactones in overcoming MAPK/ERK pathway-mediated chemotherapy resistance and warrant further investigation.

### STAT3 pathway

**Figure 11:**
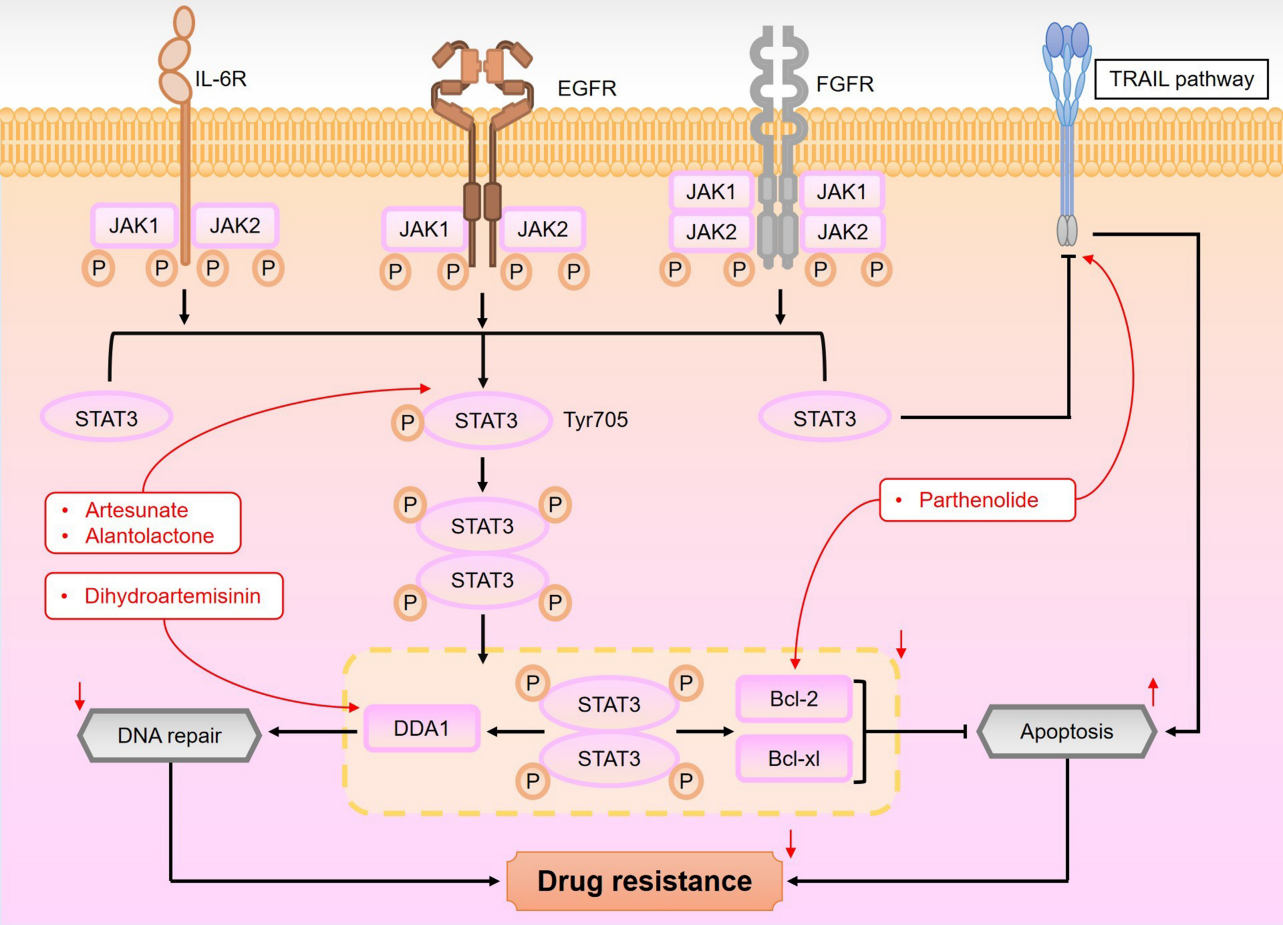
An overview of the STAT3 pathway and its role in drug resistance modulation by sesquiterpene lactones. The STAT3 pathway is activated when cytokines, such as EGF and FGF, bind to their respective receptors. This binding activates JAKs, which then phosphorylate STAT3 at tyrosine residue Tyr705, leading to its activation. Upon activation, STAT3 dimerizes and translocates to the nucleus, where it regulates gene expression. The activation of STAT3 is associated with chemotherapy resistance, as it upregulates downstream factors involved in apoptosis inhibition and DNA repair. Moreover, the STAT3 pathway interacts with the TRAIL pathway, which contributes to the ability of sesquiterpene lactones to overcome drug resistance in cancer cells. STAT3: Signal transducer and activator of the transcription 3; EGF: epidermal growth factor; FGF: fibroblast growth factor; JAKs: Janus kinases; TRAIL: tumor necrosis factor-related apoptosis-inducing ligand.

The STAT3 signaling pathway plays a pivotal role in regulating various aspects of cancer cell biology, including proliferation, survival, invasion, and immunosuppression, making it a critical target for anticancer strategies^[174,175]^. As shown in Figure 11, the STAT3 pathway is activated by cytokines such as epidermal growth factor (EGF), fibroblast growth factor (FGF), and interleukin-6 (IL-6), which bind to their respective receptors^[176,177]^. This binding activates Janus kinases (JAKs), which, in turn, phosphorylate tyrosine residues on STAT3, specifically Tyr705. The phosphorylated STAT3 then binds to these phosphotyrosines through its Src Homology 2 (SH2) domain, promoting STAT3 dimerization. These dimers translocate to the nucleus, where they bind to specific DNA response elements, initiating the transcription of genes that regulate cell growth and survival^[178,179]^.

In addition to the classical pathway, a non-classical STAT3 pathway has been identified, characterized by the phosphorylation of serine 727. This alternative activation occurs via serine/threonine kinases such as JNK1/2, MAPKs, and CDK5, and it is increasingly recognized for its role in cancer biology^[180]^. Furthermore, the subcellular localization of STAT3 plays a crucial role in regulating autophagy. Nuclear STAT3 suppresses autophagy by upregulating negative regulators like BCL2, BCL2L1, and MCL1, whereas cytoplasmic STAT3 inhibits autophagy by interacting with proteins such as EIF2AK2 and FOXO^[181,182]^.

Given its central role in tumorigenesis and resistance to therapy, STAT3 represents an important target for therapeutic intervention. Specifically, sesquiterpene lactones have shown promising potential in overcoming chemotherapy resistance by modulating the STAT3 pathway^[183]^. These compounds inhibit STAT3 signaling by either blocking its phosphorylation or targeting upstream JAK kinases, thereby preventing STAT3 activation and enhancing the effectiveness of chemotherapeutic agents^[184]^. For example, in doxorubicin-resistant breast cancer cells, upregulated STAT3 activation correlates with drug resistance. Alantolactone, a sesquiterpene lactone, significantly reduces STAT3 phosphorylation, enhancing the anti-proliferative effects of doxorubicin and overcoming resistance^[185]^. Similarly, in acute myeloid leukemia (AML), aberrant JAK/STAT3 activation contributes to drug resistance. Artesunate inhibits JAK/STAT3 signaling, reversing resistance to cytarabine and demonstrating the potential of sesquiterpene lactones in AML treatment^[186]^.

In addition to its role in drug resistance, STAT3 promotes cancer cell survival by upregulating anti-apoptotic proteins such as Bcl-2 and Bcl-xL, which help cancer cells evade chemotherapy-induced apoptosis^[187]^. Sesquiterpene lactones inhibit STAT3 activation, leading to reduced expression of these anti-apoptotic factors and enhancing tumor cell sensitivity to chemotherapy. For instance, in GC, parthenolide reverses cisplatin resistance by modulating STAT3 signaling and downregulating anti-apoptotic proteins^[66]^.

Moreover, STAT3 is critical for DNA repair mechanisms following chemotherapy-induced DNA damage. By enhancing the expression of DNA repair proteins, STAT3 contributes to cancer cell survival and resistance to treatment^[188]^. Sesquiterpene lactones, by inhibiting STAT3, impair DNA repair mechanisms, sensitizing cancer cells to chemotherapy. A notable example is dihydroartemisinin, which targets the STAT3/DDA1 pathway in breast cancer, inhibiting DDA1 expression and enhancing cisplatin efficacy^[189]^.

Persistent activation of STAT3 also contributes to resistance to TRAIL therapy by upregulating anti-apoptotic proteins and downregulating death receptor expression^[190]^. In this context, sesquiterpene lactones sensitize tumor cells to TRAIL-induced apoptosis by inhibiting STAT3. For instance, parthenolide enhances TRAIL sensitivity in HCC by inducing the expression of death receptors TRAIL-R1 and TRAIL-R2. Combining parthenolide with TRAIL significantly augments its anticancer effects and overcomes resistance^[191]^. These findings underscore the potential of sesquiterpene lactones in overcoming cancer resistance through STAT3 pathway modulation, making them promising candidates for further therapeutic development.

## DISCUSSION

In this review, we systematically analyze the chemical properties of 30 natural sesquiterpene lactones and their recent advances in anticancer and anti-drug resistance effects, focusing particularly on elucidating their mechanisms of action through five key signaling pathways. Despite the promising anticancer potential of sesquiterpene lactones, their clinical applications face several significant challenges, including low water solubility, poor stability, high toxicity, and insufficient bioavailability. These issues hinder their broader use in clinical settings. However, structural derivatization has emerged as a promising strategy to overcome these limitations. Modifying the molecular structure of sesquiterpene lactones, such as parthenolide and micheliolide, by introducing functional groups enhances their specificity for cancer cell targets, thus improving anticancer efficacy^[192]^. Additionally, synthesizing dimers or polymers, such as xanthipungolide, through a biomimetic tandem reaction using xanthatin as a precursor, has been shown to enhance both stability and biological activity^[193]^. Furthermore, combining sesquiterpene lactone derivatives with targeted delivery vectors, such as nanoparticles or antibodies, holds significant promise for improving specificity and minimizing toxicity to normal cells^[194]^. These advancements address the inherent limitations of sesquiterpene lactones and pave the way for their broader integration into clinical practice.

Expanding the scope of research, recent studies have increasingly emphasized the impact of sesquiterpene lactones on cancer stem cells (CSCs) and the immune system, further enhancing their anticancer potential. CSCs are characterized by self-renewal and differentiation capabilities, which contribute to cancer progression, recurrence, metastasis, and drug resistance^[195]^. These properties make CSCs a critical target for anticancer therapies, and sesquiterpene lactones offer a unique opportunity to inhibit their role in cancer progression. For example, parthenolide has been shown to suppress CSCs self-renewal and differentiation^[196]^, while costunolide and parthenolide induce apoptosis in CSCs by elevating intracellular ROS levels^[197,198]^. Additionally, artemisinin and gaillardin inhibit CSCs migration and invasion, thereby reducing metastasis.

Beyond their effects on CSCs, sesquiterpene lactones show promise in enhancing the effectiveness of immunotherapy, particularly immune checkpoint inhibitors. Despite the success of immunotherapy in cancer treatment, CSCs often contribute to therapy resistance^[199]^. In this context, sesquiterpene lactones, such as parthenolide, have demonstrated the ability to enhance immunotherapy efficacy. Parthenolide inhibits the NF-κB signaling pathway, reduces pro-inflammatory cytokine secretion, and mitigates the immunosuppressive tumor microenvironment^[200]^. Moreover, compounds like britanin and artesunate have been shown to downregulate the immune escape molecule PD-L1 in CSCs, facilitating the effectiveness of immune checkpoint inhibitors^[86,201]^.

Another promising strategy for maximizing the therapeutic potential of sesquiterpene lactones is combination therapy. Combining these compounds with existing anticancer drugs has attracted significant attention due to its ability to optimize therapeutic efficacy, overcome drug resistance, and improve patient outcomes^[202]^. Recent studies have revealed significant synergistic effects between sesquiterpene lactones and other chemotherapeutic agents, leading to enhanced anticancer efficacy, reduced resistance, and diminished chemotherapy-related side effects^[203,204]^. This combinational approach not only refines therapeutic regimens but also provides a robust foundation for developing safer and more effective anticancer strategies.

## CONCLUSION

This review provides a comprehensive analysis of the anticancer and anti-drug resistance properties of sesquiterpene lactones. By focusing on five key signaling pathways, we have elucidated their underlying mechanisms and therapeutic potential. Additionally, we have discussed their roles in targeting CSCs, enhancing immunotherapy, and synergizing with existing drugs to overcome drug resistance. These findings offer valuable insights into the future development of sesquiterpene lactones as innovative cancer treatments. However, for these compounds to realize their full clinical potential, future research must address challenges such as optimizing bioavailability, reducing toxicity, and developing targeted delivery systems. Continued exploration in these areas is essential for improving cancer therapy outcomes and facilitating the clinical translation of these promising compounds.

## DECLARATIONS

### Copyright

© The Author(s) 2025.
